# Case report: A 51-year-old diabetic patient with primary bilateral macronodular adrenal hyperplasia and primary hyperparathyroidism

**DOI:** 10.3389/fendo.2024.1383987

**Published:** 2024-12-16

**Authors:** Yuan Zhong, Tao Zhang, Fan Li, Yong Chen, Liwei Tong, Chenxi Xia, Dong Wei

**Affiliations:** ^1^ Department of Endocrinology, Chengdu Second People’s Hospital, Chengdu, China; ^2^ Department of Medical Imaging, Chengdu Second People’s Hospital, Chengdu, China; ^3^ Department of Pathology, Chengdu Second People’s Hospital, Chengdu, China; ^4^ Department of Urology, Chengdu Second People’s Hospital, Chengdu, China; ^5^ Department of Endocrinology, Zigong Fourth People’s Hospital, Zigong, China

**Keywords:** primary bilateral macronodular adrenal hyperplasia, primary hyperparathyroidism, type 2 diabetes mellitus, Cushing’s syndrome, multiple endocrine neoplasia type 1 (MEN 1)

## Abstract

A 51-year-old female patient with diabetes mellitus and hypertension, exhibiting poor control of blood sugar and blood pressure, was unexpectedly found to have multiple large adrenal nodules, excessive cortisol secretion, and adrenocorticotropic hormone inhibition. Cortisol levels remained unresponsive to both low-dose and high-dose dexamethasone tests, leading to a diagnosis of primary bilateral macronodular adrenal hyperplasia. Concurrently, elevated blood calcium and parathyroid hormone levels, along with 99mTc-methoxyisobutyl isonitrile (99mTc-MIBI) imaging revealing increased 99mTc-MIBI uptake in the right inferior parathyroid gland, suggest the consideration of primary hyperparathyroidism. This case is presented in light of the uncommon clinical coexistence of primary bilateral macronodular adrenal hyperplasia and primary hyperparathyroidism.

## Introduction

First described by Kirschner et al. in 1964, primary bilateral macronodular adrenal hyperplasia (PBMAH) is characterized by the presence of bilateral adrenocortical benign macronodules larger than 1 cm, often associated with varying degrees of excess cortisol secretion. PBMAH is a rare cause of endogenous Cushing’s syndrome, accounting for less than 2% of cases (1). This report highlights the uncommon clinical coexistence of PBMAH and primary hyperparathyroidism. The objective is to explore management strategies for patients presenting with both conditions.

## Case presentation

A 51-year-old female patient was admitted to the hospital due to a 5-year history of thirst, excessive drinking, excessive urination, and poor blood sugar control for the past week. She had been diagnosed with type 2 diabetes mellitus (T2DM) 5 years ago and had been using metformin, glitazone, and liraglutide to manage her blood sugar over the past year. However, in the past week, the patient’s self-measured blood sugar had increased, with a fasting blood sugar level of 8.7 mmol/L and a postprandial blood sugar level of 20 mmol/L. Additionally, she had a history of hypertension for 5 years, for which she had been taking oral medications including irbesartan, nifedipine controlled-release tablets, doxazosin, and torasemide, yet her systolic blood pressure remained greater than 130 mmHg.

Previous diagnoses included chronic kidney disease, dyslipidemia, and hyperuricemia over the past 5 years. On physical examination, her blood pressure was 151/101 mmHg, BMI 32.0 kg/m^2^, with a slightly round face but no thin skin, ecchymosis, or wide purple lines observed. Auxiliary examinations revealed a glycosylated hemoglobin level of 10.6%, a fasting blood glucose level of 11.2 mmol/L, and a fasting C peptide level of 7.27 ng/mL. Blood routine and liver function tests showed no obvious abnormalities, with a serum creatinine level of 119 µmol/L, urine protein level at 2+, and urine albumin/creatinine ratio at 268 mg/mmol. The triglyceride level was measured at 3.3 mmol/L, the LDL-C level at 2.43 mmol/L. Ambulatory blood pressure monitoring indicated a mean blood pressure of 138/87 mmHg. Color Doppler ultrasound revealed fatty liver and slight enlargement of the left atrium.

Upon admission, the patient received intensive therapy including metformin, dapagliflozin, liraglutide, and insulin. However, despite gradually increasing the insulin dosage to 48 units per day, her blood sugar remained poorly controlled. Due to the patient’s obesity and round face, hypercortisolism was suspected. A cortisol rhythm test was performed, revealing abnormal cortisol levels of 21.8 µg/dL, 13.7 µg/dL, and 15 µg/dL at 8:00, 16:00, and 24:00, respectively, and an adrenocorticotropic hormone (ACTH) level of 3.1 pg/mL at 8:00 (**Table 1**). Additionally, a chest CT scan identified mulberry-like mass shadows in bilateral adrenal areas, suggesting space-occupying lesions.

**Table 1:** Laboratory examinations before and after surgery.

	Pre-operation	10 weeks	10 months	Reference
BMI (kg/m^2^)	32.0	29.6		
HbA1C (%)	10.6	8.9	8.6	3.6-6.0
BP (mmHg)	151/101	116-122/73-79		
Cr (µmol/L)	119	113	127	41-73
Ca (mmol/L)	2.25	2.53	2.43	2.11-2.52
P (mmol/L)	1.11	1.27	1.11	0.85-1.51
PTH (pg/mL)	144-157	117	176	15-65
β-CTX (pg/mL)	446	950	1438	104-1008 (postmenopause)
t-PINP (µg/L)	27.5	98.6	151	16.27-73.87 (postmenopause)
CT (pg/mL)	9.1	5.42		<6.4
COR8:00 (µg/dL)	21.8	13.6		6.02-18.4
ACTH (pg/mL)	3.1	5.0		7.2-63.3
ODST (µg/dL)	19.3	11.6		

BMI, body mass index; HbA1C, glycated hemoglobin; BP, blood pressure; Cr, creatinine; Ca, serum calcium; P, serum phosphorus; PTH, parathyroid hormone; β-CTX, β-crosslaps; t-P1NP, total type I collagen amino-terminal extension peptide; CT, calcitonin; COR, cortisol; ACTH, adrenocorticotropic hormone; ODST, overnight suppression test with 1 mg dexamethasone.

**Figure 1:**
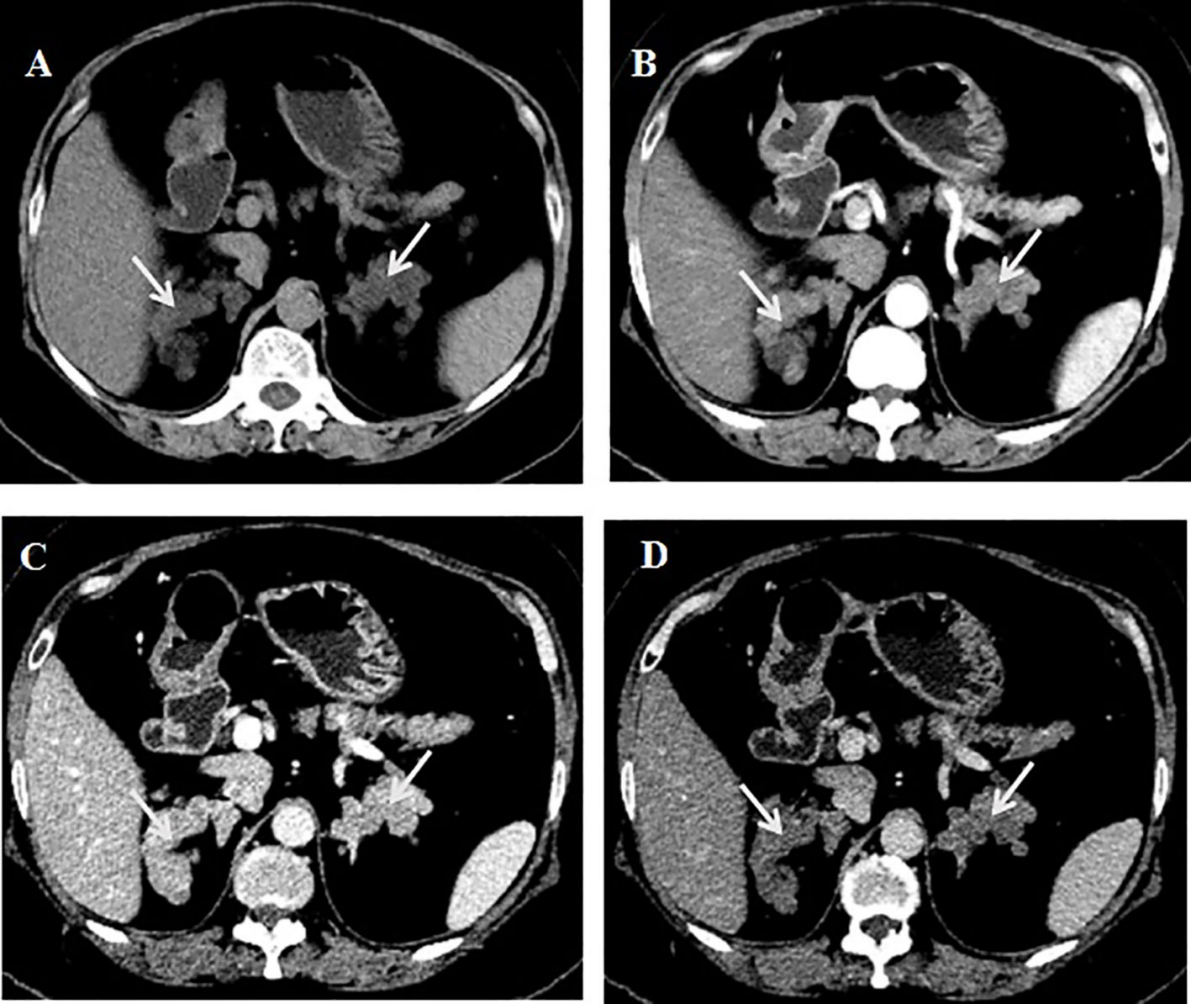
CT of adrenal gland. **(A)** Plain scan.**(B)** Enhanced scan arterial phase. **(C)** Enhanced scan portal phase. **(D)** Enhanced scan delayed phase.

A battery of tests was refined to establish the diagnosis. In an overnight suppression test with 1 mg dexamethasone, the cortisol level was 19.3 µg/dL at 8:00 after taking the medicine. In the classic low-dose dexamethasone suppression test, the cortisol level was 18.4 µg/dL at 8:00 after taking the medicine. In an overnight suppression test with 8 mg dexamethasone, the cortisol level was 17.8 µg/dL at 8:00 before and 16.6 µg/dL at 8:00 after taking dexamethasone. The prolactin level was 42.41 ng/mL (reference range: 5.18 to 26.53). The aldosterone/renin ratio and 17-hydroxyprogesterone, plasma methoxynoradrenaline and methoxyadrenaline, follicle-stimulating hormone, luteinizing hormone, growth hormone, insulin-like growth factor 1, thyroid stimulating hormone, free triiodothyronine, and free thyroxine levels were not significantly abnormal. The dehydroepiandrosterone sulfate (DHEAS) level was measured at 0.4 µmol/L (reference range: 1.5–7.7), whereas the estradiol level was recorded at 16 pg/mL (postmenopausal range: <10–28). The serum calcium level was 2.25 mmol/L, and the serum phosphorus level was 1.11 mmol/L. The 24-h urine calcium level was 3.0 mmol/L, the urine phosphorus level was 24.3 mmol/L, and the 24-h urine volume was 2.6 L. The parathyroid hormone (PTH) levels were 144 pg/mL–157 pg/mL, alkaline phosphatase level 108 U/L (reference range: 50 to 135), β-crosslap (β-CTX) level 446 pg/mL, total type I collagen amino-terminal extension peptide (t-P1NP) level 27.5 µg/L, and the 25-hydroxyvitamin D level 12.84 ng/ml. The calcitonin level was 9.1 pg/mL. Dual-energy X-ray bone mineral density suggested osteopenia. Thyroid ultrasound showed a nodule with calcification (approximately 10*10*10 mm in size) in the right lobe of the thyroid, thyroid imaging reporting and data system (TI-RADS) class 4a, and another hypoechoic nodule behind the right lobe of the thyroid, possibly derived from the parathyroid gland. 99mTc-MIBI imaging showed a low-density solid nodule behind the lower part of the right lobe of the thyroid, with clear boundaries and a size of approximately 11*9.0*8.0 mm, with increased 99mTc-MIBI uptake. CT of the adrenal gland showed multiple small nodules in the bilateral adrenal glands, some of which fused into mass shadows, with an enhanced scan showing obvious enhancement, and delayed clearance (**Figure 1**).

The patient was incidentally found to have multiple bilateral adrenal nodules with a diameter greater than 10 mm, without typical manifestations of Cushing’s syndrome. Cortisol levels were not suppressed by low and high doses of dexamethasone, and the ACTH levels decreased. Consequently, the patient was diagnosed with primary bilateral macronodular adrenal hyperplasia (PBMAH), subclinical Cushing’s syndrome, special types of diabetes, and secondary hypertension. Following thorough communication with the urologic surgeon and the patient, a right adrenalectomy was performed.

**Figure 2:**
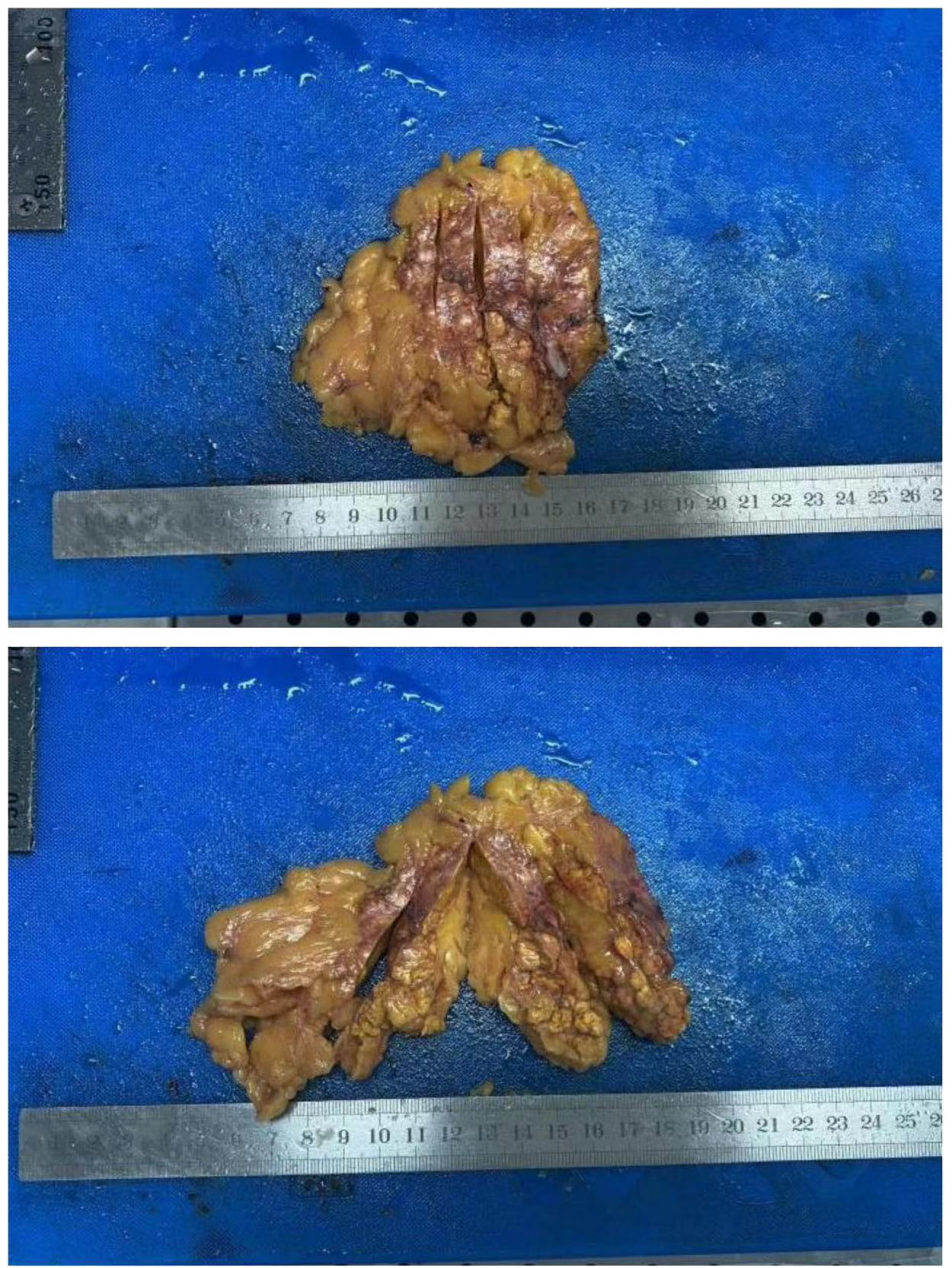
Gross specimen of the right adrenal gland.

**Figure 3:**
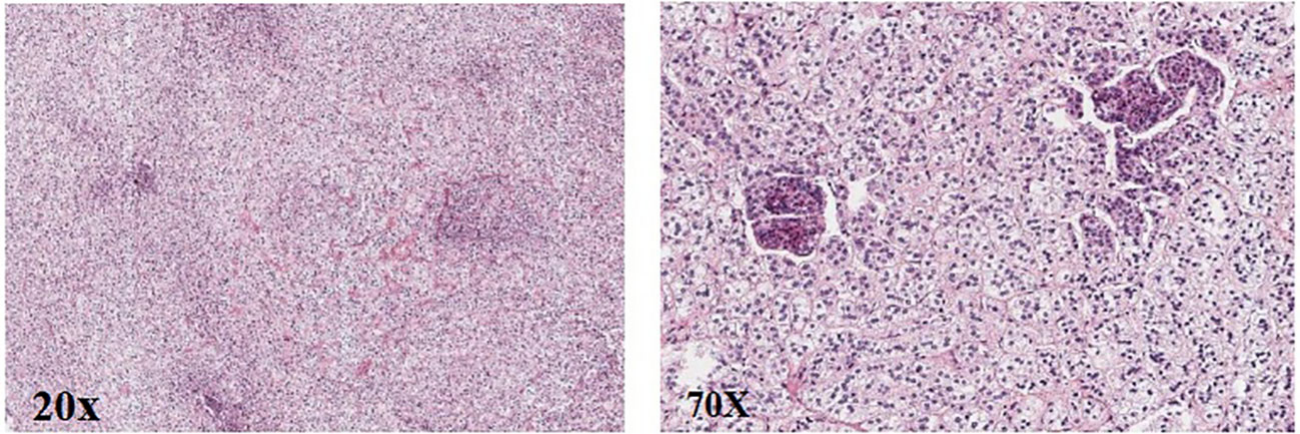
Pathology of the right adrenal gland (HE).

Post-operation, the gross specimen revealed multi-nodule fusion, measuring approximately 6.0*5.0*1.5 cm in total size, with a rough and convex surface resembling the head bun of a Buddha statue. The nodules on the section appeared yellow or golden yellow (**Figure 2**). Pathological examination at high magnification depicted cells rich in lipid, with transparent and vacuolated cytoplasm, interspersed with varying amounts of cells exhibiting dense, eosinophilic cytoplasm (**Figure 3**).

The patient was discharged from the hospital 1 week after surgery. Upon discharge, she was prescribed metformin 1.0 g, dapagliflozin 10 mg, liraglutide 1.8 mg, and insulin glargine 10 U daily to control blood glucose, as well as irbesartan 150 mg and nifedipine 30 mg daily to manage blood pressure. Regular follow-ups were scheduled.

10 weeks later, significant improvements were observed in the patient’s metabolic indexes: her weight decreased from 83.5 kg to 78.7 kg, the fasting blood glucose level stabilized around 7.0 mmol/L, postprandial blood glucose levels ranged from 7.7 mmol/L to 11.0 mmol/L, and blood pressure levels were below 130/80 mmHg. The cortisol level at 8:00 was measured at 13.6 µg/dL, with the ACTH level at 5.0 pg/mL. In an overnight suppression test with 1 mg dexamethasone, the cortisol level measured 11.6 µg/dL at 8:00 after administration. The serum calcium level was recorded at 2.53 mmol/L, PTH level at 117 pg/mL, and calcitonin level at 5.42 pg/mL. Elevated serum calcium and PTH levels were noted, and MIBI imaging showing increased uptake of 99mTc-MIBI in the posterior nodule of the right lower part of the thyroid gland, suggesting primary hyperparathyroidism and parathyroid adenoma. Given the absence of osteoporosis, urinary calculi, and other symptoms, and the slight elevation in serum calcium level, the patient underwent follow-up observation with surgery considered if necessary. During subsequent follow-up assessments, the patient exhibited progressively elevated PTH levels. 10 months post-surgery, the PTH level was measured at 176 pg/mL, whereas the creatinine levels remained relatively stable. The blood calcium concentration was recorded at 2.43 mmol/L. Additionally, the β-CTX level was 1,438 pg/ml, and the t-P1NP level was 151 µg/L.

## Discussion

Primary bilateral macronodular adrenal hyperplasia (PBMAH) is a rare cause of endogenous Cushing’s syndrome, accounting for less than 2% (1). PBMAH typically affects patients aged 40–60 years. While some patients may present with classic Cushing’s syndrome features such as thin skin, ecchymosis, broad purple lines, and decreased proximal muscle strength, most present with subclinical Cushing’s syndrome and are often diagnosed incidentally due to adrenal incidentaloma.

The pathogenesis of PBMAH involves abnormalities in hormone receptors, local ACTH production in the adrenal gland, and gene mutations. Common receptor abnormalities in PBMAH include ectopic receptor expression in the adrenal gland or increased activity of peptide hormone receptors at normal sites (2, 3). These abnormalities may involve receptors such as vasopressin, β-adrenergic receptors, luteinizing hormone/human chorionic gonadotropin receptors, serotonin receptors, glucose-dependent insulinotropic polypeptide, angiotensin receptors, G protein-coupled receptors, and their ligands. Cortisol secretion in PBMAH is partly regulated by adrenal gland-produced ACTH, leading to the abandonment of the original term “ACTH-independent macronodular adrenal hyperplasia” (4, 5). PBMAH was initially considered sporadic but is now recognized to have a genetic predisposition, inherited in an autosomal dominant manner. Genes associated with PBMAH include ARMC5, PDE11A, mutated genes of multiple endocrine adenomas type 1, and familial adenomatous polyposis, with ARMC5 being the most common mutant gene.

Laboratory examination of PBMAH typically shows varying degrees of cortisol elevation, inhibited ACTH and dehydroepiandrosterone sulfate levels, lack of cortisol suppression in low-dose and high-dose dexamethasone tests, and elevated aldosterone, 18-hydroxycorticosterone, corticosterone, and estrone levels in some patients. Imaging features include bilateral adrenal nodules of different sizes, with diameters often exceeding 10 mm, and sometimes reaching 30 mm to 40 mm, resembling ginger, mulberry, or grape clusters. Pathologically, adrenal nodules appear yellow or golden yellow on sectioning, with microscopic cells rich in lipid and vacuolar cytoplasm, mixed with varying amounts of cells with dense, eosinophilic cytoplasm, usually without pigmentation.

Diagnosis of PBMAH can be made in patients with dominant Cushing’s syndrome if ACTH is suppressed and CT indicates bilateral adrenal nodule enlargement. Additionally, PBMAH can be diagnosed if bilateral adrenal hyperplasia with large nodules is unexpectedly found, and overnight suppression test with 1 mg dexamethasone shows cortisol non-suppression. Differential diagnosis between PBMAH and primary pigmented nodular adrenocortical disease hinges on the absence of pigmentation in PBMAH nodules.

Treatment options for PBMAH patients with dominant Cushing’s syndrome or related metabolic abnormalities include surgery or drug therapy. Surgical treatment includes bilateral or unilateral adrenalectomy, with bilateral adrenalectomy reserved for severe Cushing’s syndrome cases due to resultant permanent adrenocortical hypofunction requiring lifelong hormone replacement. Unilateral adrenalectomy may be considered for patients with mild cortisol excess. The decision on which side to resect can be guided by options such as resection of the dominant side of adrenal venous sampling (AVS) cortisol levels, an estimated adrenal nodule volume model, or 131-iodine-norcholesterol (NP-59) adrenal scintigraphy. The volume of an adrenal nodule is calculated using the formula (π*a*b*c)/6, where “a,” “b,” and “c” represent the diameters of the nodule along the horizontal, sagittal, and vertical axes, respectively (6). The size of unilateral adrenal mass is determined by summing the volumes of the unilateral adrenal nodules. However, the use of AVS in PBMAH patients has not proven to be more accurate than conventional imaging, and the clinical utility of NP-59 adrenography remains constrained. It has been suggested that the best results are achieved by removing the largest adrenal gland in cases with asymmetric gland size (7). Glucocorticoid receptor antagonists and steroid synthesis inhibitors are options for patients with contraindications to surgery or mild cortisol excess. Specific receptor antagonists may also be selected based on the presence of abnormal hormone receptors (4). Previously, bilateral adrenalectomy was the standard treatment; however, recent studies have shifted toward favoring unilateral adrenalectomy or, less frequently, medical treatment with cortisol synthesis inhibitors or specific blockers of aberrant G protein-coupled receptors (7).

In this case, a 51-year-old woman with diabetes mellitus, hypertension, and low bone mass was incidentally found to have multiple large nodules in bilateral adrenal glands. Following exclusion of other causes, the diagnosis of PBMAH was established. Considering the patient’s spontaneous cortisol secretion and metabolic abnormalities, a unilateral adrenalectomy was performed. Imaging techniques were unable to differentiate the bilateral adrenal volume. The decision to excise the right adrenal gland was made due to its relatively complex anatomical position, which rendered the procedure more challenging compared with the left adrenal gland. This approach aimed to mitigate the difficulty and risk of a potential second operation should it become necessary in the future.

Although the patient’s cortisol levels were not suppressed by the 1-mg dexamethasone overnight suppression test conducted at 8:00 a.m. post-surgery, there was a reduction in her hypoglycemic and hypotensive medications following the procedure. Additionally, metabolic indicators such as blood glucose, blood pressure, and body weight showed improvement. In subsequent follow-up assessments, urinary free cortisol levels will be measured. Should elevated urinary free cortisol level be observed alongside poor metabolic control, resection of the contralateral adrenal gland will be considered.

Thyroid ultrasound revealed a calcified nodule measuring approximately 10 mm by 10 mm in the right lobe of the thyroid gland, classified as TI-RADS grade 4a. Calcitonin levels were found to range between 5.4 pg/mL and 9.1 pg/mL. Research conducted by West China Hospital indicates that the best bCtn cutoff values for the diagnosis of medullary thyroid carcinoma in the hypercalcitoninemic population with thyroid nodules are 31.54 pg/mL for men and 22.60 pg/mL for women (8). Following Chinese guidelines, we opted to monitor a patient with thyroid nodules classified as TI-RADS 4a grade with a maximum diameter of less than 15 mm.

The patient was also diagnosed with primary hyperparathyroidism. Serum calcium levels were found to be normal to slightly elevated, whereas PTH levels gradually increased without any deterioration in renal function. MIBI imaging revealed increased uptake of 99mTc-MIBI in the posterior tubercle of the lower right portion of the thyroid gland, indicative of the need for parathyroidectomy.

In patients diagnosed with primary hyperparathyroidism in conjunction with PBMAH and thyroid tumors, the possibility of multiple endocrine neoplasia type 1 should be considered. Genetic analysis or immunohistochemistry is needed to determine the underlying cause. Unfortunately, the patient did not agree with our proposal; however, this decision did not impact the treatment course. We recommend that patients consider parathyroidectomy and undergo evaluation for cortisol-related complications, as well as potential issues involving other endocrine glands. These complications include cardiometabolic disorders, bone health concerns, and neurocognitive effects.

## Data Availability

The datasets presented in this article are not readily available because of ethical and privacy restrictions. Requests to access the datasets should be directed to the corresponding author.
