# Supplementary figures and images for: Case report: A 51-year-old diabetic patient with primary bilateral macronodular adrenal hyperplasia and primary hyperparathyroidism
Source: Front Endocrinol (Lausanne). 2024 Dec 16;15:1383987. doi: 10.3389/fendo.2024.1383987 (PMC11682897; doi:10.3389/fendo.2024.1383987)

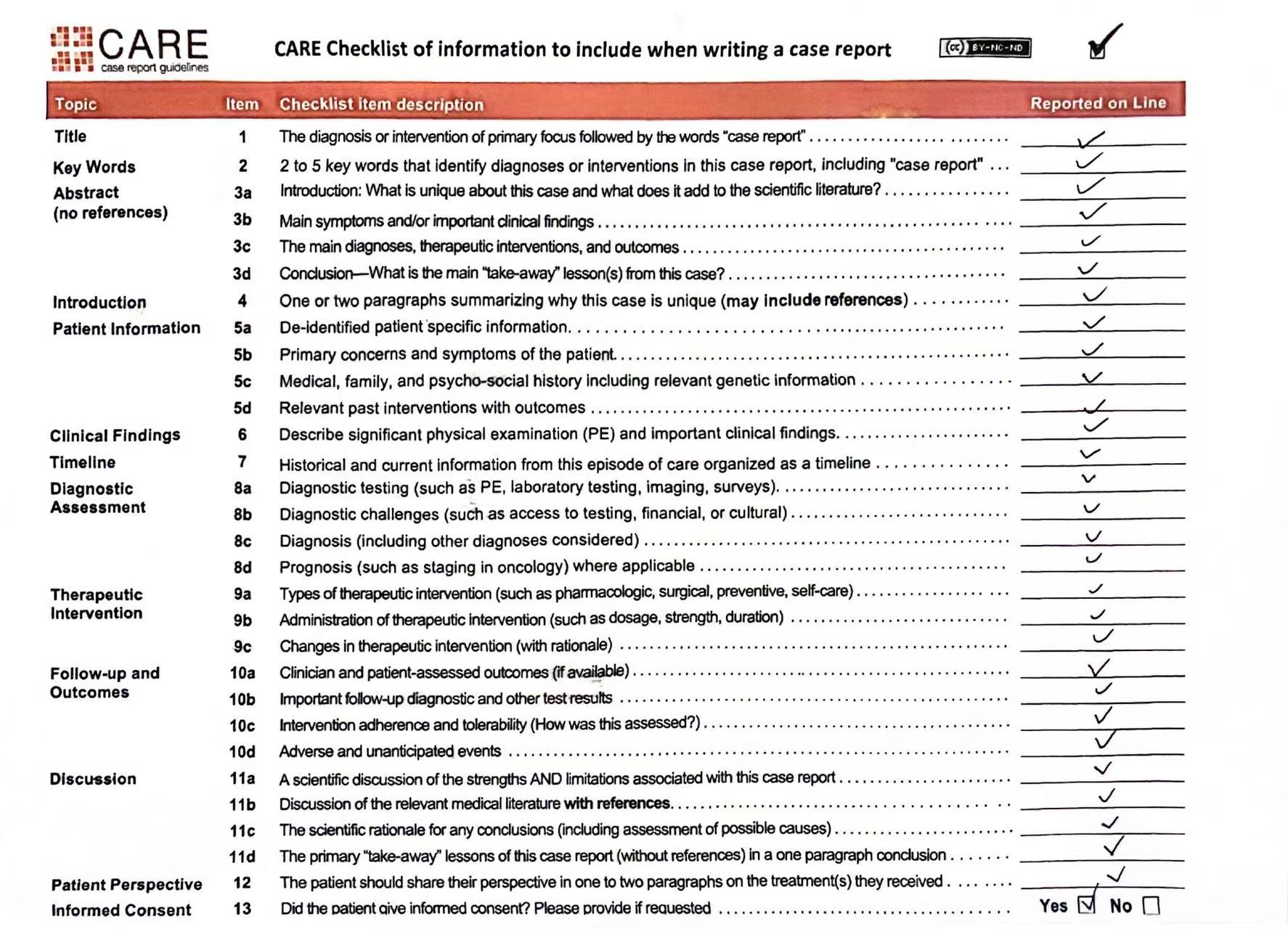

Supplement: Supplementary file 1 [file Image1.jpeg]
